# The Accumulated Clues Task (ACT): Development of a German Semantic Problem-Solving Paradigm

**DOI:** 10.5334/joc.254

**Published:** 2023-01-10

**Authors:** Charlotte S. Löffler, Sascha Topolinski

**Affiliations:** 1 Department of Psychology, University of Cologne, DE

**Keywords:** conceptual problem-solving, insight, intuition

## Abstract

The Accumulated Clues Task (ACT; Bowers et al., 1990) is a semantic problem-solving paradigm that has primarily been used in research on intuitive processes and as an experimental model of insight. In this incremental task, participants are instructed to find a solution word that is implied by a list of clue words with increasing semantic proximity to the solution word. We present a German version of the ACT, consisting of 20 word lists with 15 clues each, and report norming studies testing its psychometric properties and their relations to psycholinguistic features of the stimulus material (total *N* = 300). The results are reported and discussed for future research employing this stimulus pool, which can be easily adapted to varying experimental set-ups and research questions.

In a landmark paper that has stimulated a bulk of cognitive, social psychological, and personality research, Bowers and colleagues (1990) introduced three tasks that have since advanced our understanding of intuition and the underlying processes generating this enigmatic *feeling of knowing* (e.g., Koriat & Levy-Sadot, 2000; Metcalfe & Wiebe, 1987). Besides the Waterloo Gestalt Closure Task and the Remote Associates Test (originally adapted from Mednick, 1962), which today is a most prominent example for assessing intuitions of semantic coherence (e.g., Bolte & Goschke, 2005; Öllinger & von Müller, 2017), Bowers and colleagues presented a further language-based task, which they referred to as *the Accumulated Clues Task* (ACT). In this task, participants are presented with lists of clue words that refer to a solution word they are asked to guess.

Introduced as an experimental task assessing intuition (Bowers et al., 1990) and later framed as an experimental model of insight (Bowers et al., 1995), the ACT is an elegant model of implicit conceptual problem-solving (Reber et al., 2007). Intuition is knowing without being able to verbalize *how* one knows (e.g., Bolte & Goschke, 2005; Koriat & Levy-Sadot, 2000; Shirley & Langan-Fox, 1996; Vaughan, 1979) and insight is the abrupt realization of the solution to a cognitive problem after a period of conscious strategic study and then tacit unconscious elaboration (e.g., Bowden et al., 2005; Topolinski & Reber, 2010; Zander et al., 2016). Accordingly, participants in the ACT study the list clue for clue and incrementally narrow down the solution word by confirming hunches activated by earlier clues (Bowers et al., 1990).

Although the ACT is not a classic insight problem (see e.g., Webb et al., 2016, for a distinction between different types of insight tasks), it has indeed certain features that distinguish insight from non-insight problems (for a review of defining characteristics, see Batchelder & Alexander, 2012): (1) Each clue allows for several possible associations, however, only one of these associations leads to the solution word. (2) Most of the associations that emerge during the first clues are inadequate in that they do not lead to the correct solution word. (3) Once a productive association has emerged, it quickly leads to the solution, as this hunch can be confirmed based on the subsequent clues. (4) Solving a list involves the use of knowledge that is familiar to the solver; and (5) gaining insight into the correct solution word can be accompanied by an “Aha! experience”, which is generally considered to be an important feature of insight problems (e.g., Bowden & Jung-Beeman, 2003b; Metcalfe & Wiebe, 1987; Reber et al., 2007; Topolinski & Reber, 2010; Zander et al., 2016).

In this regard, the ACT has been used as a fruitful tool to disentangle cognitive and meta-cognitive components in intuitive problem-solving and insight and to explore their relations to broader inter-individual differences (for reviews, see Dorfman et al., 1996; Hodgkinson et al., 2008; Runco, 2014; Shirley & Langan-Fox, 1996). Constructed by Bowers et al. (1990) to examine the convergence toward the solution in intuitive problem solving, Langan-Fox and Shirley (2003), for instance, explored the relations between the performance in the ACT with personality measures of intuition. Also, Reber et al. (2007) explored the meta-cognitive role of processing style and subjective feelings of closeness to the solution as well as relations to intelligence and personality. Further, it serves as a neat conceptual model exemplifying intuition, insight, and problem-solving (e.g., Öllinger & von Müller, 2017; Zander et al., 2016).

However, although the ACT has proven to be a versatile tool that can be adapted to a wide range of research questions, to date there is no published or standardized version of this instrument. Since the original version of the ACT created by Bowers et al. (1990) remained unpublished and, to our current knowledge, is no longer accessible, future researchers would need to create their own versions (such as e.g., Reber et al., 2007). This can be a time-consuming process and also raises questions about the comparability and informative value of the results.

For this reason, we report the development and test of a German version, which can easily be modified for use in various experimental settings, for instance, as a Deese-Roediger-McDermott (DRM) paradigm (see discussion).

## Aim and Objectives of the Present Work

Our goal was to create a modern standardized version of the ACT that would enable data collection in an online setting. However, given the fact that Bowers et al. (1990) first conducted the ACT more than 30 years ago, at a time when computer-based survey instruments were still a thing of the future, we aimed to adapt the original experimental procedure according to a more modern approach. Thus, on the one hand, we wanted to simplify the original task by refining or omitting procedural elements that were no longer needed in a computer-based environment. On the other hand, we wanted to create a more economical version of the task that Bowers et al. (1990) had described as “time-consuming and somewhat frustrating” (p. 86), as well as we were interested in providing participants with a more enjoyable and fluid experience. To this end, for example, we applied shorter presentation times for the stimuli and only strongly encouraged, but did not require, participants to suggest a possible solution word after each clue.

Although we were primarily interested in constructing a standardized stimulus pool for this interesting and versatile paradigm, we also wanted to examine this pool exploratively for its specific properties. In this respect, we were interested in how different measures of participants’ solution performance would be associated with each other and how some psycholinguistic parameters of our stimuli, such as the frequency of the clues in everyday language, would influence participants’ performance in this task.

## Method

The original version of the ACT, consisting of 16 word lists with 15 clue words each, was constructed by Bowers and colleagues (1990) based on an adaption (Arthur, 1964) of the Kent-Rosanoff Word Association Test (Kent & Rosanoff, 1910). Specifically, they used responses that occurred infrequently (five times or less in 1000) in response to a particular stimulus word in that test and randomly assigned them to a position between 1 and 12 in the sequence of an ACT’s word list. For the last three clues, they used responses that occurred more frequently (more than five times in 1000) and randomly assigned them to be the thirteenth, fourteenth, or fifteenth clue of the ACT’s list.

Since we did not have access to such a pool of data in German, where the associations between words can differ drastically from those in English, we conducted a pre-study to evaluate the individual association of each clue with the solution word, respectively. This also provided us with the opportunity to increase the clues’ solution proximity more linearly throughout the final lists than was possible for Bowers et al. (1990; see discussion).

### Pre-Study

The pool of word lists was derived in the following way. To ensure proper item power for the resulting task while keeping the development effort within reasonable limit, we arbitrarily settled for 20 lists to be construed. As a first step, 20 nouns were chosen from a dictionary of the German language that fulfilled the following criteria based on our subjective evaluation: They had to be frequently used in everyday language, be neutral in valence, be relatively short in length (3 to 9 letters), did not contain umlauts or the ligature ß, and could easily be re-combined with many other German nouns into compound words. For each of these 20 solution words, a first pre-selection of 25 to 30 associated words was derived from *Word Associations Network* (https://wordassociations.net). Criteria for this pre-selection of clues were that they had to be relatively neutral in valence, they had to be familiar to the vast majority of native German speaker, and they had to be associated with the respective solution word in a way, a large percentage of native German speakers would be able to retrace — either by being a synonym or a closely related term of the solution word, by being a descriptive or circumscribing term, by being frequently used together with the solution word, or by forming established compound words with it. Associated words (i.e., clues) were mostly nouns, but could also be verbs, adjectives, and adverbs.

Each word list was then evaluated a second time by two independent raters to ensure that the former criteria were met. Clues that did not fully met the criteria according to one of these raters were discarded. Subsequently, we conducted a pre-study to assess the semantic proximity of each of the remaining clues (17 to 20 per word list) to their solution word.

Due to the number of involved stimuli, the semantic proximity between the clues and the respective solutions per list was assessed in two independent batches employing each one half of the lists (completion time of the task approx. 16 min per batch). Based on the assumption that a sample of 100 individuals is sufficient to map the smallest differences of interest for this research question (see e.g., Brysbaert, 2019; Wilson VanVoorhis & Morgan, 2007), two samples of *N* = 100 each native German speakers (Sample 1: 58 male, 40 female, 2 gender-diverse; *M*_age_ = 33, *SD*_age_ = 11; Sample 2: 64 male, 35 female, 1 gender-diverse; *M*_age_ = 28, *SD*_age_ = 8) were recruited. Participants were recruited via the Online-Access-Panel *Prolific Academic* (https://www.prolific.co) and received £2.00 compensation. After an initial briefing (see the full briefing instruction at https://osf.io/et5kb), they were presented one at a time with word pairs, consisting of one solution word and one of the associated clues. Participants were asked to rate the proximity of these words (“How close are the words CLUE and SOLUTION?”) on a scale from 0 (*not close at all*) to 10 (*extremely close*). The mean proximity ratings of each clue and the corresponding solution word can be assessed in the Appendix. Based on the mean proximity ratings of this pre-study, clues with low discriminatory power (i.e., clues whose increment of proximity to the next clue was very low) were eliminated wherever possible and the word lists were reduced to 15 clues each. The subsequent testing of the resulting stimulus pool in the classical experimental setup will be reported in the following section.

### Sample

Based on the recommendations of Brysbaert (2019) and Wilson VanVoorhis and Morgan (2007; see the *Pre-study* section), *N* = 100 native German speakers (60 male, 40 female; *M*_age_ = 31, *SD*_age_ = 11) were recruited via *Prolific Academic* and received £3.75 compensation.

### Materials and Procedure

The experiment was conducted in an online setting employing the software application *Inquisit Web* (2016); we will provide the script for the experiment upon direct request. Participants were informed that the experiment investigated fundamental cognitive processes of language processing. They were instructed that they were going to be presented with lists of words and would, one at a time, see all words of these lists successively. In each word list, there would be exactly one solution word that would be implied by all of the clues with increasing semantic closeness to the solution word over the course of presenting the list. Their task would be to retrieve the solution word as soon as possible by typing in a suggestion for the solution word after each clue. It would not matter if they could not think of a potential solution word once, but they should try to always suggest a word if possible, just following their first impulse. The precise briefing instruction (original German version and English translation) can be assessed at https://osf.io/et5kb.

After the instruction, the 20 word lists (see the Appendix) were presented in random order and re-randomized anew for each participant. Before the presentation of each list in the experimental block, participants were informed that they were about to start with a new word list. Then, in ascending order of semantic proximity to the solution word, each clue of a list was presented on the screen for 2,000 ms.^1^ To standardize the presentation procedure, all clues were presented in capital letters and their height was set to 8% of the screen. After the presentation of a given clue, a text box appeared and participants were prompted to enter a possible candidate for the solution word. In contrast to the procedure of Bowers et al. (1990), participants were able to skip the text box but were instructed to type in a potential solution word whenever possible. When the correct solution to a word list was given, a message appeared that the list had been solved successfully. Then, before the presentation of the next list, participants were once again informed that they were about to start with a new word list. They also received this message when the solution word was not generated after the 15^th^ clue. At the end of the experimental block, participants completed some demographic questions and were asked if they had experienced any technical problems during the experiment. The average completion time for the experiment was 34 minutes.

## Results

According to the procedure of Bowers et al. (1990), the solution performance for each list was operationalized by the number of clues needed by the participant to generate the solution word for a given list; this variable being called *clues required* hereafter. If participants were not able to generate the solution word by the 15^th^ clue, the list was scored as non-solved.^2^ We only classified lists as solved if participants produced the preordained solution word. Although it must be assumed that there are alternative solutions that make meaningful reference to most of the clues in a list, the existence of alternative solutions that relate to *all* 15 clues in this list appears highly implausible. This is because the clues not exclusively imply the solution word by being closely related terms, but also by alluding to common idiomatic expressions or by forming existing compound words with it. Therefore, based on the criterion that the solution had to be meaningfully associated with all clues in a list, only the solution word itself, its plural and verb forms, and misspellings of the solution word were accepted as correct.

**Figure 1:**
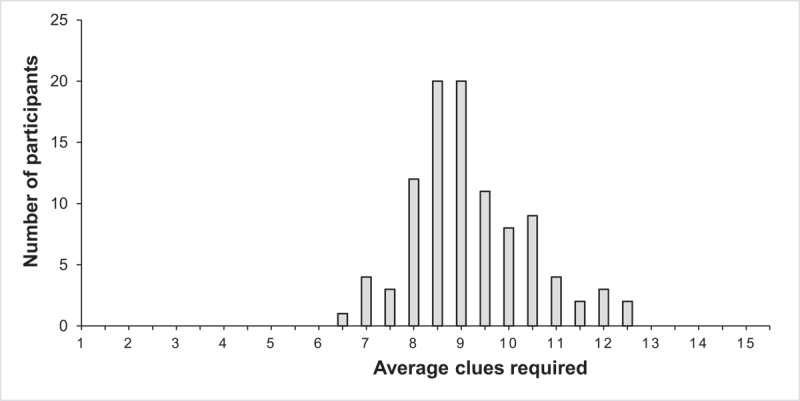
Average clues required across the sample. *Note*: *N* = 100.

The averaged psycholinguistic parameters for each list are presented in Table 1 in addition to the normative data from the present experiment. Across all word lists, an average of *M* = 9.21 (*SD* = 1.26) clues were required to generate the solution word for a given list and *M* = 0.88 (*SD* = 0.03) potential solution words were proposed per clue.^3^ There was a reasonable amount of variance in the average performance (i.e., clues required) across the sample (see Figure 1). In addition, there was sufficient variance in the lists’ relative solution difficulty, ranging from an average clues required of *M* = 4.97 (*SD* = 3.38) for the list FABRIK (FACTORY) to a clues required of *M* = 12.15 (*SD* = 2.35) for the list PAAR (PAIR); see Table 1. Overall, *M* = 66% (*SD* = 22%) of the participants solved a list with a maximum of 15 clues. We will refer to this variable as *solving probability* hereafter. The internal consistency (Cronbach’s Alpha) of clues required and the word lists’ solving probability was α = 0.83 and α = 0.84, respectively, which is superior to the α = 0.70 reported by Bowers et al. (1990).

**Table 1:** Mean clues required, solving probabilities, proposed solutions per clue, clue length, clue frequency in everyday language, and global solution proximity of the word lists.


	WORD LIST	CLUES REQUIRED *(SD)*	SOLVING PROBABILITY	PROPOSED SOLUTIONS	CLUE LENGTH	CLUE FREQUENCY	SOLUTION PROXIMITY

1	Fabrik	4.97 *(3.38)*	58%	0.90	8.07	11.67	7.03

2	Bach	5.52 *(2.58)*	61%	0.93	6.00	12.73	6.43

3	Boot	6.77 *(2.73)*	84%	0.92	5.87	12.40	6.70

4	Marmor	6.92 *(2.96)*	65%	0.87	5.27	13.13	6.09

5	Zug	7.95 *(3.34)*	95%	0.90	6.87	11.87	6.55

6	Mund	8.40 *(3.39)*	83%	0.92	6.07	11.87	6.80

7	Lamm	8.47 *(2.96)*	55%	0.88	5.53	12.73	5.72

8	Silber	9.09 *(3.22)*	58%	0.90	6.07	13.00	6.54

9	Adler	9.32 *(2.57)*	77%	0.88	6.40	12.67	5.91

10	Treppe	9.34 *(2.35)*	91%	0.88	6.73	12.40	5.84

11	Glas	9.71 *(3.18)*	90%	0.86	6.80	12.67	6.38

12	Draht	9.78 *(2.81)*	49%	0.86	6.93	14.40	5.57

13	Nacht	10.11 *(3.25)*	83%	0.88	5.40	11.07	6.32

14	Schachtel	10.34 *(2.54)*	70%	0.88	7.47	12.80	5.88

15	Kuppel	10.36 *(3.77)*	42%	0.88	7.40	12.60	6.20

16	Mantel	10.44 *(3.00)*	61%	0.88	5.33	11.67	5.60

17	Salbe	10.75 *(4.06)*	73%	0.89	6.40	14.00	6.60

18	Punkt	11.32 *(4.26)*	31%	0.81	5.33	9.53	5.04

19	Knoten	11.79 *(3.32)*	48%	0.85	7.40	12.47	5.42

20	Paar	12.15 *(2.35)*	47%	0.87	6.20	12.07	5.42

	**Total mean**	**9.21 *(1.26)***	**66%**	**0.88**	**6.38**	**12.39**	**6.10**


*Note*: *N* = 100. Clues required (with standard deviations in parentheses) indicate the average number of clues required by the participants to generate the solution word for the given list of clues. Solving probability indicates the percentage of participants who were able to generate the solution word with a maximum of 15 clues. Proposed solutions indicates the average number of proposed solution words per clue. Clue length indicates the average number of letters of the lists’ clues. Clue frequency denotes the global frequency of the lists’ clues in everyday language corpora (retrieved from https://corpora.uni-leipzig.de) with higher values denoting lower frequency. Solution proximity indicates the average semantic proximity of all clues of a list to the solution word of that list (data from pre-study).

### Exploratory Analyses

The present rich performance data of 100 participants allowed further in-depth analyses of certain performance parameters and their relations to the psycholinguistic features of the word lists. We held no particular hypotheses regarding those detailed relationships but examined them exploratively and report them here to stimulate future hypothesis development regarding driving cognitive mechanisms of the performance in this task. Table 2 presents the exploratory item-based correlation analysis among these list parameters.

**Table 2:** Bivariate correlations among list parameters.


	1	2	3	4	5	6	7	8

1. Clues required ^a^	—							

2. Solving probability	–0.30	—						

3. Proposed solutions	–0.66**	0.49*	—					

4. Clue length	–0.03	0.00	0.05	—				

5. Clue frequency	–0.09	0.12	0.28	0.28	—			

6. Solution frequency	–0.03	–0.11	0.05	0.22	0.66**	—		

7. First clue frequency	–0.28	–0.47*	–0.02	0.08	0.21	0.44*	—	

8. Solution proximity	–0.67**	0.56*	0.79**	0.21	0.19	0.02	0.02	—


*Note*: *N* = 20. ^a^ Lower clues required indicate higher (faster) solution performance. * *p* < 0.05, ** *p* < 0.01.

**Figure 2:**
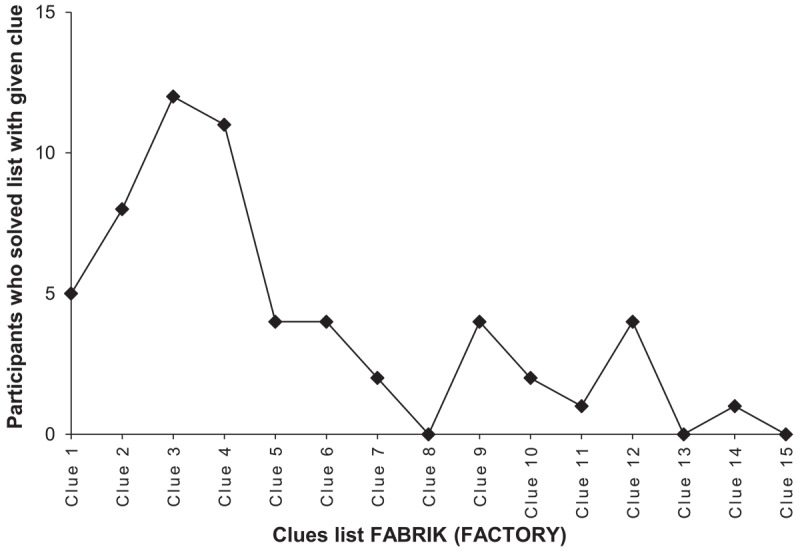
Frequencies of clues required for the list FABRIK (FACTORY). *Note*: Number of participants who found the correct solution word with the respective clue over the course of the list FABRIK (FACTORY). *N*_solved_ = 58.

**Figure 3:**
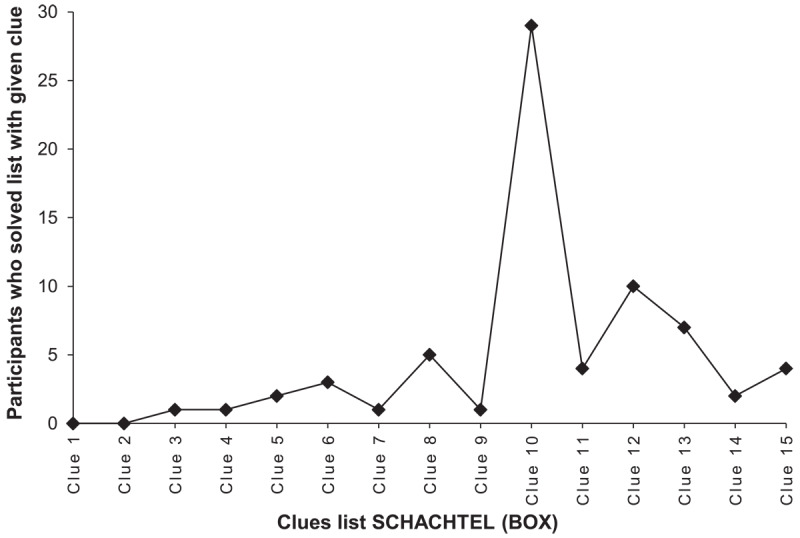
Frequencies of clues required for the list SCHACHTEL (BOX). *Note*: Number of participants who found the correct solution word with the respective clue over the course of the list SCHACHTEL (BOX). *N*_solved_ = 70.

First of all, intriguingly, within the present stimulus pool of 20 word lists, clues required and solving probability were not significantly correlated with each other, *r* = –0.30, *p* = 0.197. That is, the number of clues that participants required to solve the lists was unrelated to the proportion of participants who solved the lists at all. For instance, the list FABRIK (FACTORY) required the least clues for those participants who solved it, but its solving probability is comparatively low. Analyzing the frequencies of clues required for each list separately, we found that most lists featured one (or sometimes two) *critical spots* regarding the function between clues required and solving probability. These critical spots occur at certain sequences of clues (e.g., SCHLOT [CHIMNEY] – BACKSTEIN [BRICK] – KONZERN [CORPORATION], the first clues of the list FABRIK [FACTORY]) that seem to interact with each other and coalesce into a particularly strong intuitive hunch that increases the probability of solving the lists at these spots. At the same time, as illustrated in Figures 2 and 3 with the examples of the lists FABRIK (FACTORY) and SCHACHTEL (BOX), the solving probability tends to drastically decrease when the lists are not solved at these critical spots, which we deem as an explanation for the non-significant correlation between clues required and solving probability. We tentatively interpret these fascinating junctures as critical transition phases in the semantic representational space of a given list, which should be further explored in the future.

Further, the number of proposed solutions per clue correlated moderately to strongly with the clues required, *r* = -0.66, *p* = 0.001; as well as the solving probability, *r* = 0.49, *p* = 0.028. That is, the more potential solution candidates participants proposed, the faster they found the actual solution word and the higher their chance of solving the list was, although their solution propositions were not evaluated by feedback.

Clue length in letters did not relate substantially to any of the other measures (all *p*s > 0.213). We had only included clue length since it is a commonly controlled stimulus feature in research on accumulative semantic priming (e.g., Topolinski & Strack, 2009a, b, c) and a possible determinant of semantic fluency (e.g., Topolinski & Reber, 2010).

The frequencies of the clues and solutions in everyday language (retrieved from https://corpora.uni-leipzig.de; higher values mean lower frequency) yielded only the significant relationship that the more frequent the first clue of a given list was the more probably that list was solved across all participants (solving probability), *r* = –0.47, *p* = 0.034. However, the frequency of the first clue did not substantially relate to clues required, *r* = –0.28, *p* = 0.226; and neither frequency of the whole list nor the frequency of the solution determined any solution outcome (all *p*s > 0.225). These null findings are at odds with the literature on word frequency and semantic priming, where high frequent words generate stronger priming (McNamara, 2005). This whole pattern is particularly striking given the fact that the only significant predictor was the frequency of the first clue, the clue farthest away from the solution in (list) space and (trial) time. This might be interpreted in the way that the first clue, when relatively familiar, increases the motivation or attention to engage with a given list in the first place (for similar effects in emotion processing accuracy, see Elfenbein & Ambady, 2003).

Regarding the semantic relationship between the list and its solution, the global proximity of the clues to their solution correlated moderately to strongly with clues required, *r* = –0.67, *p* = 0.001; the solving probability, *r* = 0.56, *p* = 0.010; and the number of proposed solution words, *r* = 0.79, *p* < 0.001, respectively. That is, the closer a list as a whole was to its solution semantically, the fewer clues were needed for solution, the more attempts were made to find the solution word, and the more participants solved a list eventually, which is in accordance with the literature on semantic relatedness and insight (e.g., Bowden & Jung-Beeman, 2003a; Bowden et al., 2005; Topolinski & Reber, 2010).

## Discussion

A German version of the Accumulated Clues Task (Bowers et al., 1990) was constructed and its psycholinguistic properties were normed on *N* = 300 native German-speaking participants. We consider our version to be a modern replica of the instrument developed by Bowers et al. (1990), which unfortunately is no longer accessible. Since we are the first to publish a standardized stimulus pool for this task, our goal is to make it available to other researchers along with the normative information and psycholinguistic parameters of this instrument.

Our German version demonstrated appropriate variance in the solution difficulty of the individual lists and the solution performance of the participants. In addition, the present stimulus pool has a good internal consistency of α = 0.83, which is higher compared to the version of Bowers and colleagues, however, this is most likely because our pool contains 20 lists instead of 16 (cf., Bowers et al., 1990).

Regarding the number of clues required to generate the correct solution word, our pool of lists appears to be slightly more difficult to solve, but fairly comparable to the original one by Bowers and colleagues. Accordingly, our participants arrived at the correct solution word with an average of *M* = 11.39 (*SD* = 1.95) clues,^4^ compared to the *M* = 10.12 (*SD* = 4.55) clues reported by Bowers et al. (1990).

Apart from our main goal to construct a standardized stimulus pool for this interesting and versatile paradigm, we also wanted to examine this pool exploratively for its specific properties. The exploratory correlational analyses among the lists’ properties yielded the following interesting results. First, the number of clues required to solve a given list did not significantly correlate with the general probability that this list was solved at all in the present sample. Rather, most lists happened to feature critical sequences of clues (at varying junctures across the lists) that phasically boosted solution likelihood. When participants did not retrieve the correction solution at these sweet spots, overall probability of finding the solution decreased drastically. We can only speculate about the nature of these sweet spots. Possibly, at these junctures in the list the clues were effective to critically re-organize the multi-dimensional semantic space (cf., e.g, Rodd, 2020) of the cognitive representation of the list prompting its common semantic denominator. When participants did not retrieve the solution at this juncture, they might have deviated into testing remote semantic hypotheses that were not supported by the further upcoming clues, or lost motivation in engaging with the list at all. Future research should further explore this most interesting finding.

Second, neither the global word frequency of the list nor that of the solution determined solution performance, as one would expect from the literature on semantic priming (e.g., McNamara, 2005; Plaut & Booth, 2000), but selectively the word frequency of the first clue determined the eventual solving of a given list. Yet again, this frequency of the first clue did not substantially correlate with clues required to solve the list. This suggests a rather motivational than automatic spreading mechanism: The familiarity of the very first clue triggered motivation to engage with the list and find its solution (see for familiarity and stimulus encoding depth, Elfenbein & Ambady, 2003). If an automatic spreading route would have taken course, first-clue frequency would have (negatively) correlated with clues required, since the high-frequent first clues would have activated solution-related information more efficiently than low-frequent first clues. Crucially, future studies might actively manipulate the familiarity or fluency of the first clue (e.g., by pre-exposure) to test this experimentally.

Third, the semantic solution proximity of the list as a whole determined solution performance. This is perfectly in line with the literature on semantic priming showing that close semantic relations generate higher priming effects than distant relations (e.g., Kiefer et al., 1998; McNamara & Healy, 1988; Spitzer et al., 1993; Topolinski & Deutsch, 2013).

As stated in the previous sections, we opted for a slight adaption of the original experimental procedure to create a more modern, economic, and less frustrating task. Let us briefly consider how each of these adaptions may have affected the general quality of our task. Our experimental procedure differs from that of Bowers and colleagues (1990) mainly in four aspects:

First, in our version of the ACT, the solution proximity of the clues increases more linearly to the solution word, whereas in the original version, the first 12 clues possess equally low solution proximity, while the last three clues are more closely associated with the solution word. It is conceivable that our linear progression towards the solution leads to a slightly slower insight into the solution word, however, given the similar average clues required reported by Bowers et al. (1990), this seems rather negligible.

Second, in contrast to the original procedure, we strongly encouraged, but did not require, participants to suggest a possible solution word after each clue. Considering the nevertheless satisfactory rate of *M* = 0.88 proposed solutions per clue, this should not represent a limitation of our procedure either.

Third, compared to the original procedure in which the first clue was presented for 15 seconds and the presentation intervals for subsequent clues became progressively shorter, down to a minimum of 10 seconds (cf., Bowers et al., 1990), we applied drastically shorter presentation intervals of 2 seconds per clue (for demonstrations of the speed of semantic coherence discrimination and insight problem-solving, see e.g., Bolte & Goschke, 2005; Bowden & Jung-Beeman, 2003b). The advantage of this adaption is corroborated by our results, which show that participants were nevertheless able to solve the lists with a similar number of clues as in the original procedure.

Fourth, in contrast to our present procedure, Bowers et al. (1990) additionally asked participants to indicate promising solutions but to continue the lists until they were convinced that their solution was correct. The fact that we did not implement this additional step is mainly due to the different objectives of our research. Whereas Bowers et al. (1990) aimed to study the process of gaining insight into the solution, we were primarily interested in constructing a standardized stimulus pool that could be adapted to a wide range of research questions. In this regard, our task can easily be modified to explore procedural aspects of intuitive problem-solving and insight, for example, by instructing participants to indicate potentially promising solutions and to decide for themselves when to declare the lists satisfactorily solved.

### Implementation in different areas of research

The present stimulus pool is not only a versatile tool in the field of intuitive problem-solving and insight but could also be modified for other areas of cognitive research. For instance, the stimulus architecture (not the task) of the ACT resembles the well-known Deese-Roediger-McDermott (DRM) paradigm. In this paradigm assessing the emergence of false memories, participants are shown a list of words that are all associated with a common denominator that is itself not presented (Roediger & McDermott, 1995). As a result, in later memory probing participants erroneously report having seen the implied but not presented common denominator (e.g., Storbeck & Clore, 2005; Van Damme, 2013; for reviews, see Roediger et al., 1998; Schacter, 1999). Although previous researchers have produced a number of German DRM lists (e.g., Stegt, 2006), to our current knowledge these are not publicly available and the pool of lists is limited. To avoid the time-consuming process of creating new German DRM lists when a larger number of stimuli is required, the present stimulus pool also offers the possibility of modification into a DRM set-up. The only difference between the ACT and the DRM (except participants’ task) is that the clues are presented in increasing semantic relatedness to the solution in the ACT, while there is no such incremental semantic determination in the DRM (which is why DRM lists cannot be converted to ACT lists, but only the other way around). This can easily be modified by presenting the present clues in random order and probing later memory.

## Conclusion

In conclusion, the present report provides a standardized German version of the ACT for use in future research that can be easily adapted to varying experimental set-ups and research questions. For instance, future research can explore the impact of psychological variables such as cognitive mind-sets, creativity inductions, mood, and motivation selectively on easy and hard ACT items. Also, already the present explorative analyses brought about novel research avenues, such as the role of familiarity of the initial clue or the appearance of semantic sweet spots along a given list that foster or inhibit eventual problem solving—both phenomena to be further explored.

## Data Accessibility Statement

Data and materials are available at https://osf.io/et5kb.

## Additional File

The additional file for this article can be found as follows:

10.5334/joc.254.s1 Appendix. Accumulated Clues Task (ACT, German version).

## Footnotes

This differs from the procedure of Bowers et al. (1990), in which the clues were shown for a minimum of 10,000 ms. each. However, after extensively testing the presentation intervals of the clues ourselves and also presenting the experiment to several independent testers, we concluded that two seconds were sufficient to process each clue (for demonstrations of the speed of semantic coherence discrimination and insight problem-solving, see e.g., Bolte & Goschke, 2005; Bowden & Jung-Beeman, 2003b) while keeping participants as engaged as possible.

This is in contrast to the original scoring procedure of Bowers et al. (1990), who assigned a clues required of 16 to non-solved lists and did not report the proportion of solved lists as a separate measure, which, however, we consider appropriate for a differentiated interpretation of the results.

This high proportion of solutions attempts results from the fact that participants were instructed to suggest a solution word whenever possible, even at the beginning of a list (see the *Materials and Procedure* section).

The average clues required reported here was calculated according to the slightly different scoring procedure of Bowers et al. (1990), who assigned a clues required of 16 to non-solved lists.
