# Supplementary material for: The Accumulated Clues Task (ACT): Development of a German Semantic Problem-Solving Paradigm
Source: J Cogn. 2023 Jan 10;6(1):3. doi: 10.5334/joc.254 (PMC9838243; doi:10.5334/joc.254)
Supplement: Appendix. — Accumulated Clues Task (ACT, German version). [file joc-6-1-254-s1.pdf]

Appendix

Accumulated Clues Task (ACT, German version)

Table 1

Word lists of the ACT, each denominated after their solution word (English translation in parentheses), with the clues' mean semantic proximity to the solution word (pre-study)

**List 1:**	**FABRIK (FACTORY)**	
	**Clues**	$M_{\text{Proximity}}$
Clue 1	SCHLOT	4.12
Clue 2	BACKSTEIN	4.21
Clue 3	KONZERN	6.02
Clue 4	GELÄNDE	6.39
Clue 5	SCHICHT	6.64
Clue 6	HERSTELLER	6.73
Clue 7	BETRIEB	7.11
Clue 8	MECHANISCH	7.31
Clue 9	FERTIGUNG	7.49
Clue 10	HALLE	7.85
Clue 11	MASCHINE	8.05
Clue 12	FLIEßBAND	8.16
Clue 13	ARBEITER	8.18
Clue 14	PRODUKTION	8.36
Clue 15	INDUSTRIE	8.79
**List 2:**	**BACH (STREAM)**	
	**Clues**	$M_{\text{Proximity}}$
Clue 1	SCHMAL	3.81
Clue 2	BRÜCKE	5.02
Clue 3	UFER	5.08
Clue 4	MÜNDEN	5.26
Clue 5	QUELLE	5.72
Clue 6	LAUF	5.73
Clue 7	PLÄTSCHERN	5.84
Clue 8	SCHILF	5.95

Clue 9	BAROCK	6.61
Clue 10	GEWÄSSER	6.96
Clue 11	DAMM	7.37
Clue 12	ORGEL	7.55
Clue 13	KOMPONIST	8.32
Clue 14	MÜHLE	8.61
Clue 15	FLUSS	8.65
**List 3:**	**BOOT (BOAT)**	
	**Clues**	$\boldsymbol{M}_{\textbf{Proximity}}$
Clue 1	SCHAUKELN	4.81
Clue 2	KURS	5.20
Clue 3	SCHLAUCH	6.10
Clue 4	WRACK	6.24
Clue 5	KENTERN	6.33
Clue 6	BUG	6.35
Clue 7	MOTOR	6.40
Clue 8	MARINE	6.53
Clue 9	SCHWIMMEN	6.54
Clue 10	ANKER	7.01
Clue 11	RUDER	7.29
Clue 12	FISCHER	7.30
Clue 13	SEGEL	7.57
Clue 14	MEER	8.00
Clue 15	SCHIFF	8.78
**List 4:**	**MARMOR (MARBLE)**	
	**Clues**	$\boldsymbol{M}_{\textbf{Proximity}}$
Clue 1	ERLESEN	3.61
Clue 2	ADER	3.96
Clue 3	KALK	4.19
Clue 4	MASERUNG	5.20
Clue 5	GRAB	5.29
Clue 6	BÜSTE	5.37
Clue 7	MEIßEL	5.42
Clue 8	SÄULE	6.47

Clue 9	WEIß	6.63
Clue 10	PLATTE	6.80
Clue 11	GLATT	6.99
Clue 12	KUCHEN	7.09
Clue 13	HART	7.58
Clue 14	STATUE	7.74
Clue 15	STEIN	8.99
**List 5:**	**ZUG (TRAIN)**	
	**Clues**	$M_{\text{Proximity}}$
Clue 1	VOGEL	2.17
Clue 2	LINIE	4.63
Clue 3	FAHRZEUG	4.68
Clue 4	RESERVIEREN	5.19
Clue 5	GÜTER	5.83
Clue 6	KLASSE	5.96
Clue 7	ANBINDUNG	6.00
Clue 8	REISE	6.40
Clue 9	PASSAGIER	6.70
Clue 10	ABTEIL	7.64
Clue 11	SITZPLATZ	7.70
Clue 12	LOK	8.25
Clue 13	SCHAFFNER	8.74
Clue 14	WAGGON	9.13
Clue 15	BAHNHOF	9.27
**List 6:**	**MUND (MOUTH)**	
	**Clues**	$M_{\text{Proximity}}$
Clue 1	BEFEUCHTEN	4.80
Clue 2	TROCKEN	4.88
Clue 3	BART	5.20
Clue 4	KOPF	5.32
Clue 5	VOLL	5.72
Clue 6	ATMUNG	5.94
Clue 7	ÖFFNUNG	6.21
Clue 8	MIMIK	6.53

Clue 9	SCHLUCKEN	7.25
Clue 10	KIEFER	7.57
Clue 11	KAUEN	7.81
Clue 12	ZÄHNE	8.48
Clue 13	SPRECHEN	8.58
Clue 14	ESSEN	8.78
Clue 15	LIPPEN	8.98
**List 7:**	**LAMM (LAMB)**	
	**Clues**	$\boldsymbol{M}_{\textbf{Proximity}}$
Clue 1	SCHMOREN	3.37
Clue 2	KIND	3.57
Clue 3	FRÜHLING	3.64
Clue 4	ZART	4.67
Clue 5	KEULE	4.90
Clue 6	UNSCHULD	5.78
Clue 7	WEIß	5.82
Clue 8	OSTERN	5.82
Clue 9	FELL	6.00
Clue 10	KOTELETT	6.35
Clue 11	JUNG	6.43
Clue 12	WEIDE	6.72
Clue 13	WOLLE	6.97
Clue 14	HIRTE	7.33
Clue 15	SCHAF	8.40
**List 8:**	**SILBER (SILVER)**	
	**Clues**	$\boldsymbol{M}_{\textbf{Proximity}}$
Clue 1	ANLAUFEN	4.22
Clue 2	SPIEGEL	4.32
Clue 3	FISCH	5.30
Clue 4	SCHIMMERN	5.36
Clue 5	ERZ	5.73
Clue 6	LEGIERUNG	6.01
Clue 7	GLANZ	6.38
Clue 8	MINE	6.64

Clue 9	MÜNZE	6.85
Clue 10	SCHMIED	7.21
Clue 11	GOLD	7.71
Clue 12	MEDAILLE	7.76
Clue 13	EDEL	7.91
Clue 14	SCHMUCK	8.23
Clue 15	METALL	8.42
**List 9:**	**ADLER (EAGLE)**	
	**Clues**	$\boldsymbol{M}_{\textbf{Proximity}}$
Clue 1	STEIN	3.05
Clue 2	WIPFEL	3.12
Clue 3	SEE	4.03
Clue 4	WIND	4.16
Clue 5	SCHWINGEN	5.18
Clue 6	SYMPBOL	5.31
Clue 7	KREISEN	5.49
Clue 8	SPANNWEITE	6.01
Clue 9	HORST	6.09
Clue 10	WAPPEN	6.40
Clue 11	KRALLEN	7.00
Clue 12	AUGE	7.51
Clue 13	SCHNABEL	7.87
Clue 14	FEDER	8.45
Clue 15	GREIFVOGEL	9.01
**List 10:**	**TREPPE (STAIRCASE)**	
	**Clues**	$\boldsymbol{M}_{\textbf{Proximity}}$
Clue 1	PODEST	3.53
Clue 2	HALLE	3.54
Clue 3	KNARREN	3.83
Clue 4	BETRETEN	4.43
Clue 5	LEITER	4.83
Clue 6	STEIL	4.96
Clue 7	KELLER	5.67
Clue 8	FAHRSTUHL	5.83

Clue 9	WENDEL	6.38
Clue 10	AUFGANG	6.55
Clue 11	ABSATZ	6.85
Clue 12	HINUNTER	6.92
Clue 13	STOCKWERK	7.40
Clue 14	GELÄNDER	7.80
Clue 15	STUFE	9.15
**List 11:**	**GLAS (GLASS)**	
	**Clues**	$\boldsymbol{M}_{\textbf{Proximity}}$
Clue 1	KNOCHEN	3.83
Clue 2	SCHLEIFEN	4.71
Clue 3	SPRUNG	5.15
Clue 4	QUARZ	5.34
Clue 5	HAUS	5.38
Clue 6	GLANZ	5.47
Clue 7	SAND	5.64
Clue 8	SPLITTERN	6.26
Clue 9	KRISTALL	6.39
Clue 10	SPIEGEL	6.89
Clue 11	BRILLE	7.43
Clue 12	TRANSPARENT	7.82
Clue 13	SCHERBE	8.23
Clue 14	FENSTER	8.49
Clue 15	SCHEIBE	8.63
**List 12:**	**DRAHT (WIRE)**	
	**Clues**	$\boldsymbol{M}_{\textbf{Proximity}}$
Clue 1	DURCHBRENNEN	3.12
Clue 2	SCHLINGE	3.94
Clue 3	ESEL	3.99
Clue 4	WICKELN	4.09
Clue 5	GLÜHEN	4.41
Clue 6	STACHEL	5.35
Clue 7	BÜRSTE	5.84
Clue 8	LÖTEN	6.15

Clue 9	ELEKTRIZITÄT	6.18
Clue 10	MASCHEN	6.27
Clue 11	KUPFER	6.43
Clue 12	BIEGSAM	6.57
Clue 13	LEITUNG	6.92
Clue 14	ZAUN	6.96
Clue 15	METALL	7.39
**List 13:**	**NACHT (NIGHT)**	
	**Clues**	$\boldsymbol{M}_{\textbf{Proximity}}$
Clue 1	FEUER	3.08
Clue 2	MORGEN	4.55
Clue 3	NEBEL	4.65
Clue 4	LAGER	4.71
Clue 5	WACHE	4.82
Clue 6	DIENST	5.27
Clue 7	KÜHL	5.78
Clue 8	TAG	6.12
Clue 9	EULE	6.85
Clue 10	DÄMMERUNG	6.97
Clue 11	RUHE	7.42
Clue 12	BETT	7.89
Clue 13	STERN	8.21
Clue 14	SCHLAF	8.91
Clue 15	DUNKELHEIT	9.52
**List 14:**	**SCHACHTEL (BOX)**	
	**Clues**	$\boldsymbol{M}_{\textbf{Proximity}}$
Clue 1	KASSETTE	2.99
Clue 2	HOLZ	3.24
Clue 3	SCHUH	4.28
Clue 4	GESCHENK	4.92
Clue 5	INHALT	5.19
Clue 6	SCHATULLE	5.72
Clue 7	ÖFFNEN	6.03
Clue 8	AUFBEWAHREN	6.15

Clue 9	BEHÄLTER	6.29
Clue 10	ZIGARETTE	6.47
Clue 11	KARTON	6.88
Clue 12	PRALINEN	6.99
Clue 13	STREICHHOLZ	7.51
Clue 14	VERPACKUNG	7.63
Clue 15	BOX	7.96
**List 15:**	**KUPPEL (DOME)**	
	**Clues**	$\boldsymbol{M}_{\textbf{Proximity}}$
Clue 1	ÜBERSPANNEN	3.68
Clue 2	DURCHMESSER	4.74
Clue 3	BOGEN	4.81
Clue 4	MOSCHEE	5.35
Clue 5	GEWÖLBE	6.02
Clue 6	GEBÄUDE	6.07
Clue 7	HOCH	6.29
Clue 8	DECKE	6.30
Clue 9	KATHEDRALE	6.31
Clue 10	ARCHITEKTUR	6.62
Clue 11	WÖLBEN	6.64
Clue 12	PLANETARIUM	6.77
Clue 13	RUND	7.72
Clue 14	HALBKUGEL	7.76
Clue 15	DOM	7.87
**List 16:**	**MANTEL (COAT)**	
	**Clues**	$\boldsymbol{M}_{\textbf{Proximity}}$
Clue 1	KABEL	2.94
Clue 2	ROCK	3.03
Clue 3	REIFEN	3.31
Clue 4	HÜLLE	3.77
Clue 5	UNIFORM	3.95
Clue 6	FUTTER	5.08
Clue 7	KNOPF	5.16
Clue 8	PELZ	5.71

Clue 9	WOLLE	5.88
Clue 10	KRAGEN	6.16
Clue 11	JACKE	7.25
Clue 12	MODE	7.38
Clue 13	LANG	7.56
Clue 14	KLEIDUNG	8.13
Clue 15	WINTER	8.76
**List 17:**	**SALBE (OINTMENT)**	
	**Clues**	$\boldsymbol{M}_{\textbf{Proximity}}$
Clue 1	HEXE	2.81
Clue 2	TALG	3.42
Clue 3	TINKTUR	4.90
Clue 4	JUCKEN	5.72
Clue 5	REIBEN	6.10
Clue 6	VERBRENNUNG	6.30
Clue 7	FETT	6.77
Clue 8	WIRKSTOFF	6.85
Clue 9	LINDERN	7.25
Clue 10	ARZNEI	7.43
Clue 11	HAUT	7.84
Clue 12	HEILEN	7.98
Clue 13	APOTHEKE	8.18
Clue 14	AUFTRAGEN	8.60
Clue 15	CREME	8.81
**List 18:**	**PUNKT (POINT/DOT)**	
	**Clues**	$\boldsymbol{M}_{\textbf{Proximity}}$
Clue 1	WUND	2.12
Clue 2	THEMA	2.92
Clue 3	FLECK	3.24
Clue 4	ORT	3.61
Clue 5	PLATZIERUNG	3.76
Clue 6	ZIEL	4.00
Clue 7	ZEIT	4.24
Clue 8	LINIE	4.40

Clue 9	STRICH	5.21
Clue 10	LANDUNG	5.71
Clue 11	STOPP	5.88
Clue 12	GENAU	5.93
Clue 13	ZEICHEN	7.80
Clue 14	ENDE	7.99
Clue 15	KOMMA	8.75
**List 19:**	**KNOTEN (KNOT)**	
	**Clues**	$M_{\text{Proximity}}$
Clue 1	ZUNGE	3.35
Clue 2	BRUST	3.45
Clue 3	ACHT	4.12
Clue 4	VERSCHLUNGEN	4.22
Clue 5	GESCHWINDIGKEIT	4.75
Clue 6	HAAR	4.82
Clue 7	DOPPELT	5.21
Clue 8	MATROSE	5.26
Clue 9	ZUSAMMENZIEHEN	5.48
Clue 10	KRAWATTE	5.60
Clue 11	FEST	5.70
Clue 12	SCHLEIFE	6.34
Clue 13	SCHLAUFE	7.28
Clue 14	BINDEN	7.47
Clue 15	SEIL	8.18
**List 20:**	**PAAR (PAIR)**	
	**Clues**	$M_{\text{Proximity}}$
Clue 1	STORCH	2.33
Clue 2	ALLEIN	2.53
Clue 3	HOSE	3.44
Clue 4	LAUF	3.98
Clue 5	KIND	4.46
Clue 6	HUF	4.66
Clue 7	TRENNUNG	5.08
Clue 8	BRAUT	5.42

Clue 9	ZWILLING	6.00
Clue 10	ZUEINANDER	6.15
Clue 11	TANZ	6.18
Clue 12	EHE	7.35
Clue 13	ZUSAMMENGEHÖRIG	7.72
Clue 14	SOCKEN	7.91
Clue 15	PARTNER	8.15
